# Effects of ticagrelor in a mouse model of ischemic stroke

**DOI:** 10.1038/s41598-017-12205-w

**Published:** 2017-09-21

**Authors:** Keita Yamauchi, Takahiko Imai, Masamitsu Shimazawa, Toru Iwama, Hideaki Hara

**Affiliations:** 1 Molecular Pharmacology, Department of Biofunctional Evaluation, Gifu Pharmaceutical University, Gifu, 501-1196 Japan; 2 Department of Neurosurgery, Gifu University Graduate School of Medicine, Gifu, 501-1194 Japan

## Abstract

Ticagrelor is a direct acting and reversibly binding P2Y12 antagonist approved for the prevention of thromboembolic events. Its potential benefits in ischemic stroke have not been investigated sufficiently. Mice were subjected to 2 hours of transient middle cerebral artery occlusion (MCAO). Mice were orally treated with ticagrelor (10 or 30 mg/kg), aspirin (60 mg/kg), or vehicle at 3 and 24 hours before MCAO and 0 and 6 hours after reperfusion. The infarct volume and neurological deficits 22 hours after reperfusion were evaluated. Cerebral blood flow (CBF) within 24 hours after MCAO was monitored. We performed western blotting and *in vitro* analysis using oxygen-glucose deprivation (OGD) stress in human brain microvessel endothelial cells (HBMVECs) to investigate the protective effects of ticagrelor. Ticagrelor (30 mg/kg) improved neurological deficits, reduced the infarct volume, and improved CBF. It promoted the phosphorylation of endothelial nitric oxide synthase (eNOS) and extracellular signal-regulated kinase 1/2 (ERK1/2) during the early phase after reperfusion. Increased phosphorylation of eNOS and ERK1/2 were also observed in HBMVECs after OGD stress. Ticagrelor attenuate ischemia reperfusion injury possibly via phosphorylation of eNOS and ERK1/2 in endothelial cells. This suggests that ticagrelor has neuroprotective effects via mechanisms other than its antiplatelet action.

## Introduction

Stroke is one of the most common causes of death and disability worldwide^1^. In patients at high risk for occlusive vascular events, including myocardial infarction, ischemic stroke, angina and peripheral arterial disease, antiplatelet therapy is a key treatment to prevent the including myocardial infarction, ischemic stroke, angina and peripheral arterial disease including myocardial infarction, ischemic stroke, angina and peripheral arterial disease. Ticagrelor is an oral P2Y12 receptor antagonist with a distinct mechanism of action compared with the thienopyridines (clopidogrel, prasugrel and ticlopidine). Unlike the thienopyridines which are all prodrug with active metabolites binding irreversibly ticagrelor is direct acting and reversibly binding. In the Platelet Inhibition and Patient Outcomes trial (PLATO), which is a phase III trial in patients with acute coronary syndrome (ACS), ticagrelor reduced the composite endpoint of cardiovascular death, myocardial infarction, and stroke when compared to clopidogrel without increasing overall major bleeding^2^. Based on the clinical evidence, ticagrelor has been approved for the prevention of thromboembolic events in ACS patients. Although the benefit of ticagrelor is likely primarily related to the antiplatelet effect, additional mechanisms have been proposed^3^. Several authors have reported that ticagrelor inhibits the cellular uptake of adenosine. Armstrong *et al*. reported that ticagrelor inhibits the cellular uptake of adenosine by inhibition of equilibrative nucleoside transporter-I (ENT-1)^4^. In rat and pig ischemia reperfusion models, chronic treatment with ticagrelor reduced myocardial infarct volume via an adenosine-dependent mechanism^5^. These reports implicate that ticagrelor has other effects than P2Y12 inhibition.

Although ticagrelor is expected to provide benefit in the settings of cerebral ischemia, experimental and clinical evidence are limited^6^. In an experimental study using a permanent rat middle cerebral artery occlusion (MCAO) model, Gelosa *et al*. reported that the neuroprotective effects of ticagrelor occur via P2Y12-mediated inhibition of microglial activation^7^.

To investigate the additional effects of ticagrelor, we investigated the effects of ticagrelor against cerebral ischemia reperfusion injury (IRI) *in vivo* using an experimental murine transient MCAO model and *in vitro* using an oxygen-glucose deprivation (OGD) model of human brain microvascular endothelial cells (HBMVECs).

## Results

### Effects of Ticagrelor and Aspirin on Infarct Volume and Neurological Deficits

**Figure 1:**
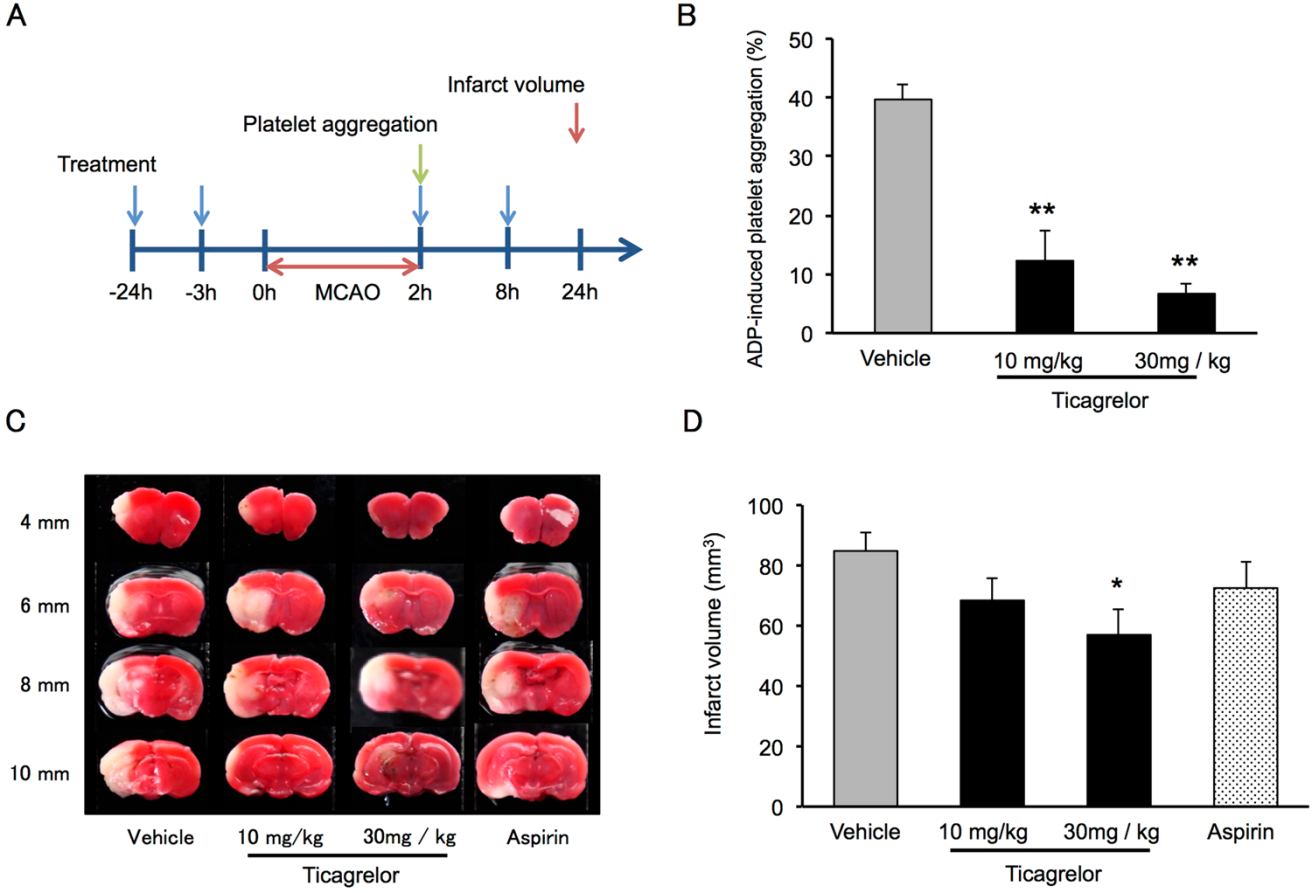
Effects of ticagrelor on platelet aggregation and infarct volume 22 hours after reperfusion. (**A**) Experimental protocol for the measurement of platelet aggregation and infarct volume. (**B**) Adenosine diphosphate-induced platelet aggregation after administration of ticagrelor. Data are expressed as means ± standard errors of the mean (SEMs). N = 3 or 4 per group; **P < 0.01 vs. vehicle; Student’s *t*-test. (**C**) Representative images of triphenyltetrazolium chloride-stained coronal slices 22 hours after reperfusion. (**D**) Quantitative measurements of infarct volume 22 hours after reperfusion. Data are expressed as means ± SEMs. N = 10 to 12 per group; *P < 0.05 vs. vehicle; Student’s *t*-test.

The protocol used in this experiment is illustrated in Fig. 1A. Adenosine diphosphate (ADP)-induced platelet aggregation after oral intake of ticagrelor is shown in Fig. 1B. Ticagrelor significantly inhibited platelet aggregation at both 10 and 30 mg/kg. Representative images from the 2,3,5-triphenyltetrazolium chloride (TTC) staining experiment and measurements of infarct volume indicate that ticagrelor at 30 mg/kg significantly reduce the infarct volume 22 hours after reperfusion when compared to vehicle treatment (Fig. 1C,D).

**Figure 2:**
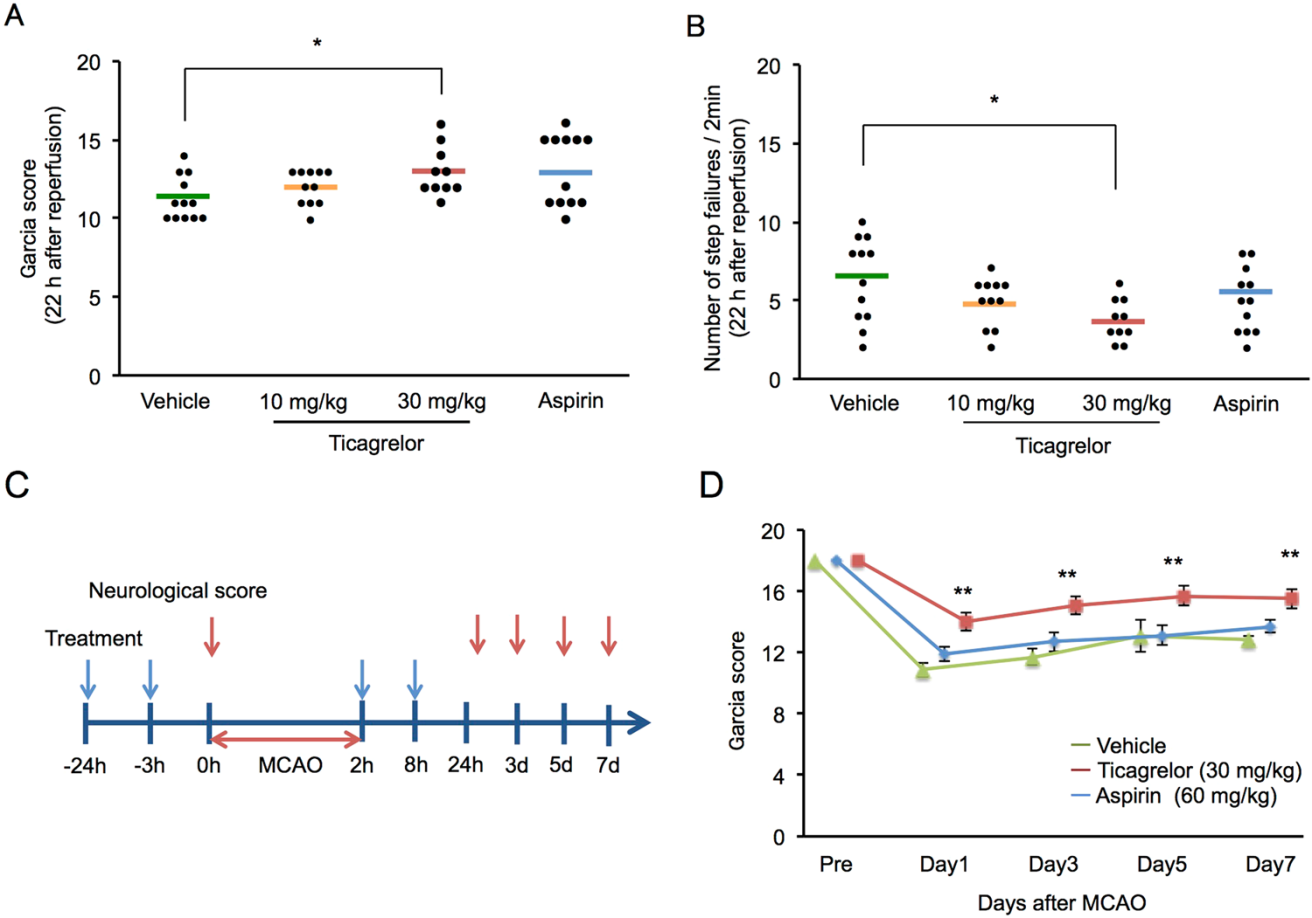
The effects of ticagrelor on neurological deficits. (**A**) Neurological deficits 22 hours after reperfusion measured using Garcia score. (**B**) Neurological deficits 22 hours after reperfusion measured using the grid walk test. N = 10 to 12 per group; *P < 0.05 vs. vehicle; Wilcoxon signed-rank test. (**C**) The experimental protocol used to measure the neurological score. (**D**) The results of Garcia scores within 7 days after ischemia. N = 10 to 12 per group; **P < 0.01 vs. vehicle; Wilcoxon signed-rank test.

Ticagrelor at 30 mg/kg significantly improved neurological deficits as measured using the Garcia score (Fig. 2A) and the grid-walk test (Fig. 2B) 22 hours after reperfusion. We also monitored the Garcia score up to 7 days after the MCAO, during which time the protective effects of ticagrelor were maintained (Fig. 2C,D).

### Effects of Ticagrelor on Cerebral Blood Flow during Ischemia Reperfusion

**Figure 3:**
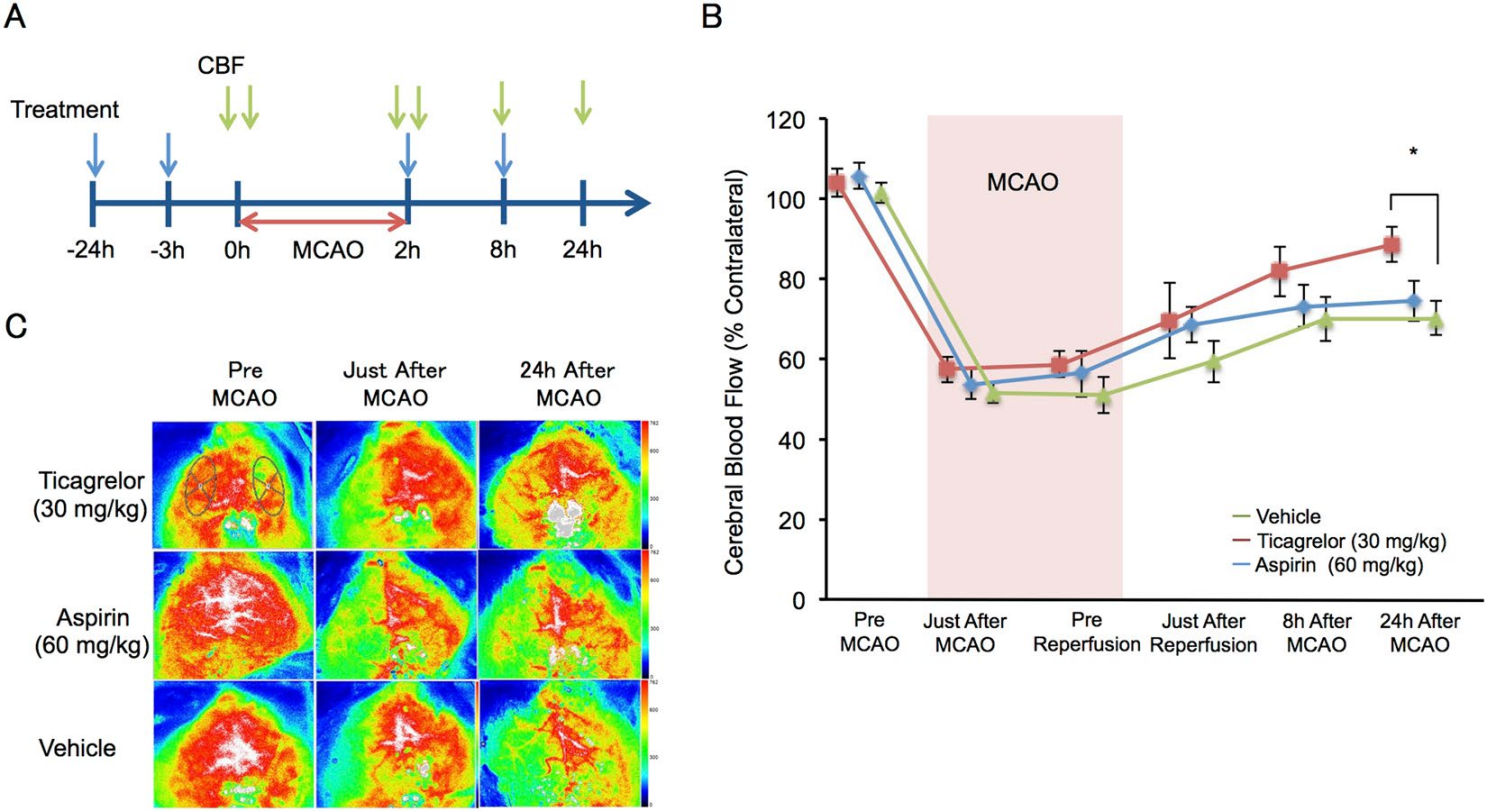
The effects of ticagrelor on cerebral blood flow (CBF), as measured using laser speckle Doppler. (**A**) The experimental protocol used for the CBF measurements. (**B**) Quantitative data of CBF within 24 hours after reperfusion. Data are expressed as means ± standard errors of the mean. N = 7 to 9 per group; *P < 0.05 vs. vehicle; Student’s *t-*test. (**C**) Representative images of CBF. The oval area in the image obtained from ticagrelor-treated mice before middle cerebral artery occlusion indicates the region of interest.

Figure 3A shows the experimental protocol used to assess cerebral blood flow (CBF). CBF in the 30 mg/kg ticagrelor group gradually improved after reperfusion and was significantly improved 24 hours after reperfusion (Fig. 3B,C).

### Mechanisms Underlying the Protective Effects of Ticagrelor

**Figure 4:**
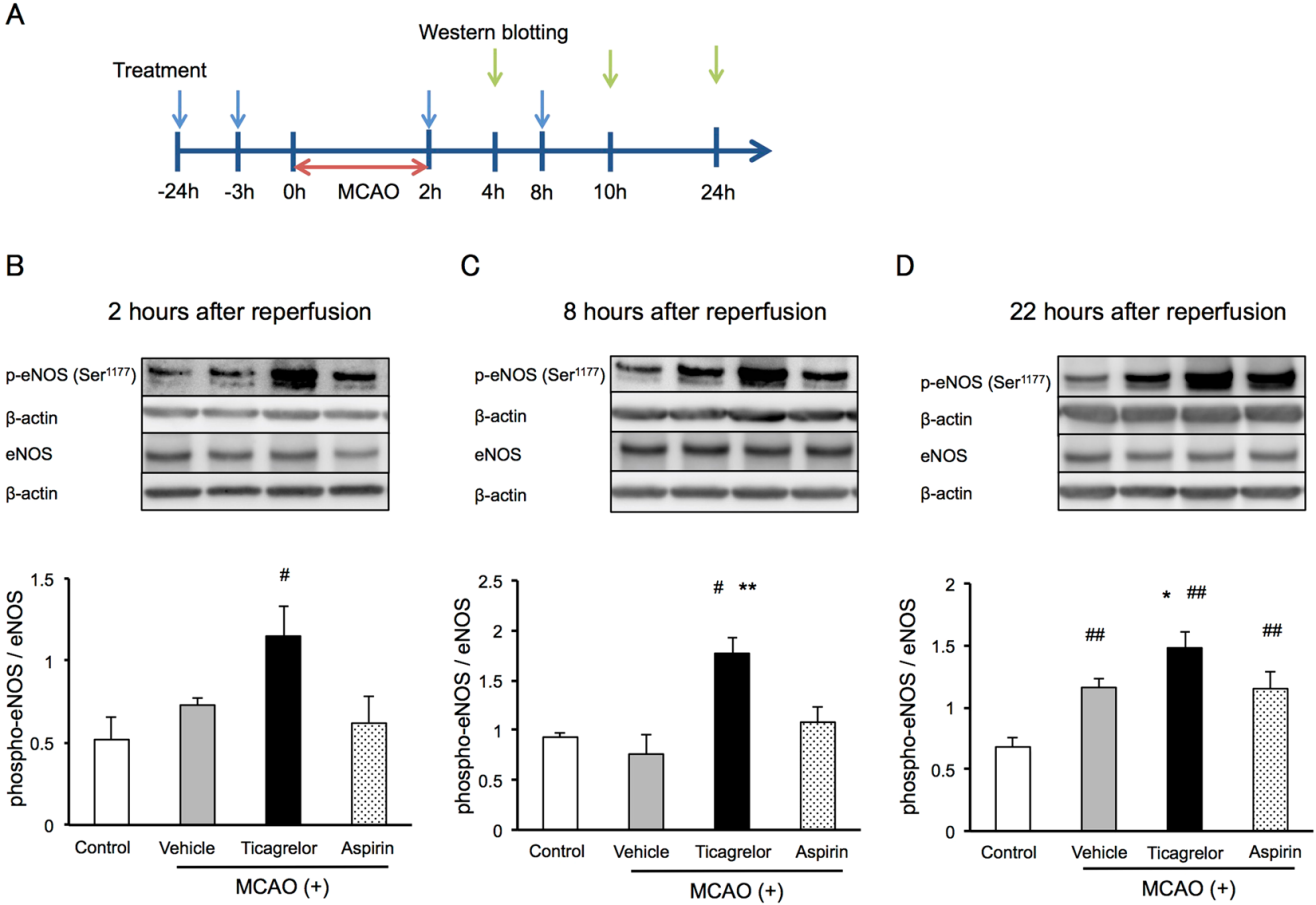
The effects of ticagrelor on phosphorylation of endothelial nitric oxide synthase (eNOS) after reperfusion. (**A**) The protocol used for western blotting. The effects of ticagrelor on the phosphorylation of endothelial nitric oxide synthase 2 hours (**B**), 8 hours (**C**), and 22 hours (**D**) after reperfusion. Data are expressed as means ± standard errors of the mean. N = 5 or 6 per group; ^#^P < 0.05, ^##^P < 0.01 vs. control, *P < 0.05 vs. vehicle; Student’s *t*-test. The cropped blots are used in this Figure and the full-length blots are presented in Supplementary Figure S1.

**Figure 5:**
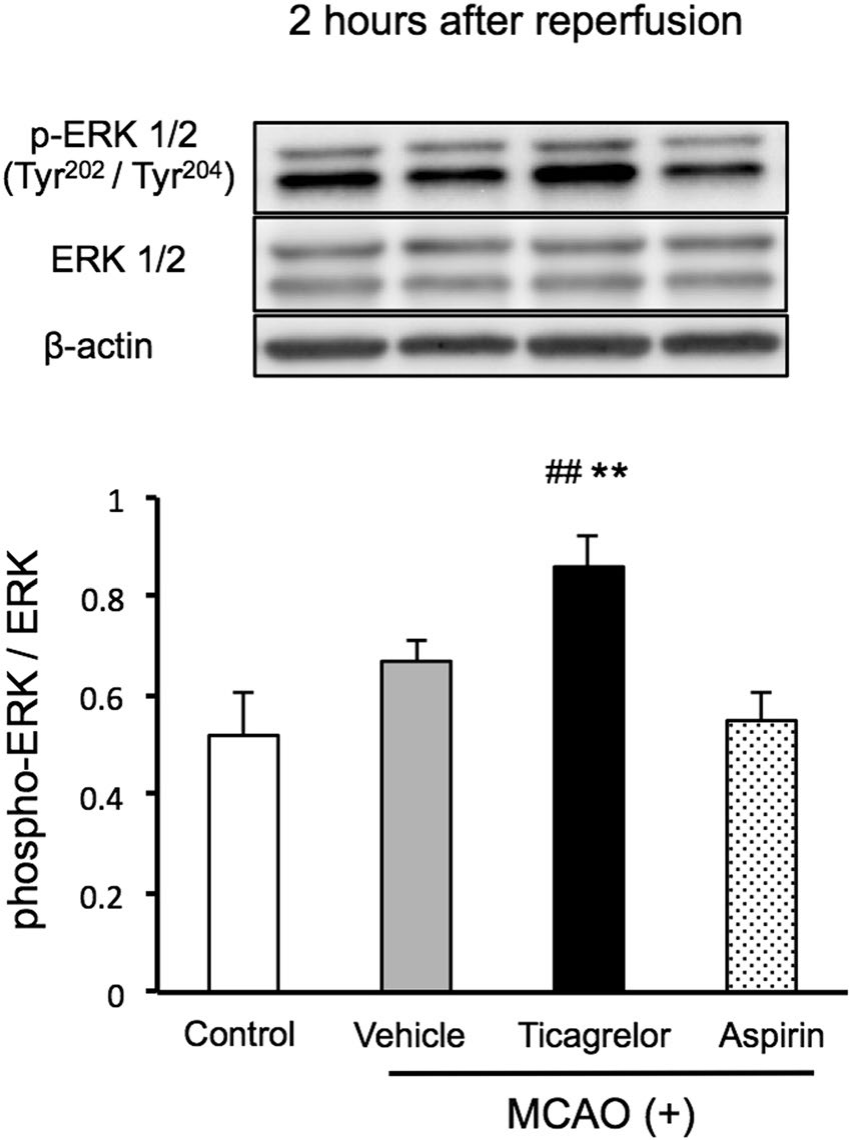
The effects of ticagrelor on the phosphorylation of extracellular signal-regulated kinase 1/2 two hours after reperfusion. Data are expressed as means ± standard errors of the mean. N = 5 or 6 per group; **P < 0.01 vs. control, ^#^P < 0.05 vs. vehicle; Student’s *t*-test. The cropped blots are used in this Figure and the full-length blots are presented in Supplementary Figure S2.

**Figure 6:**
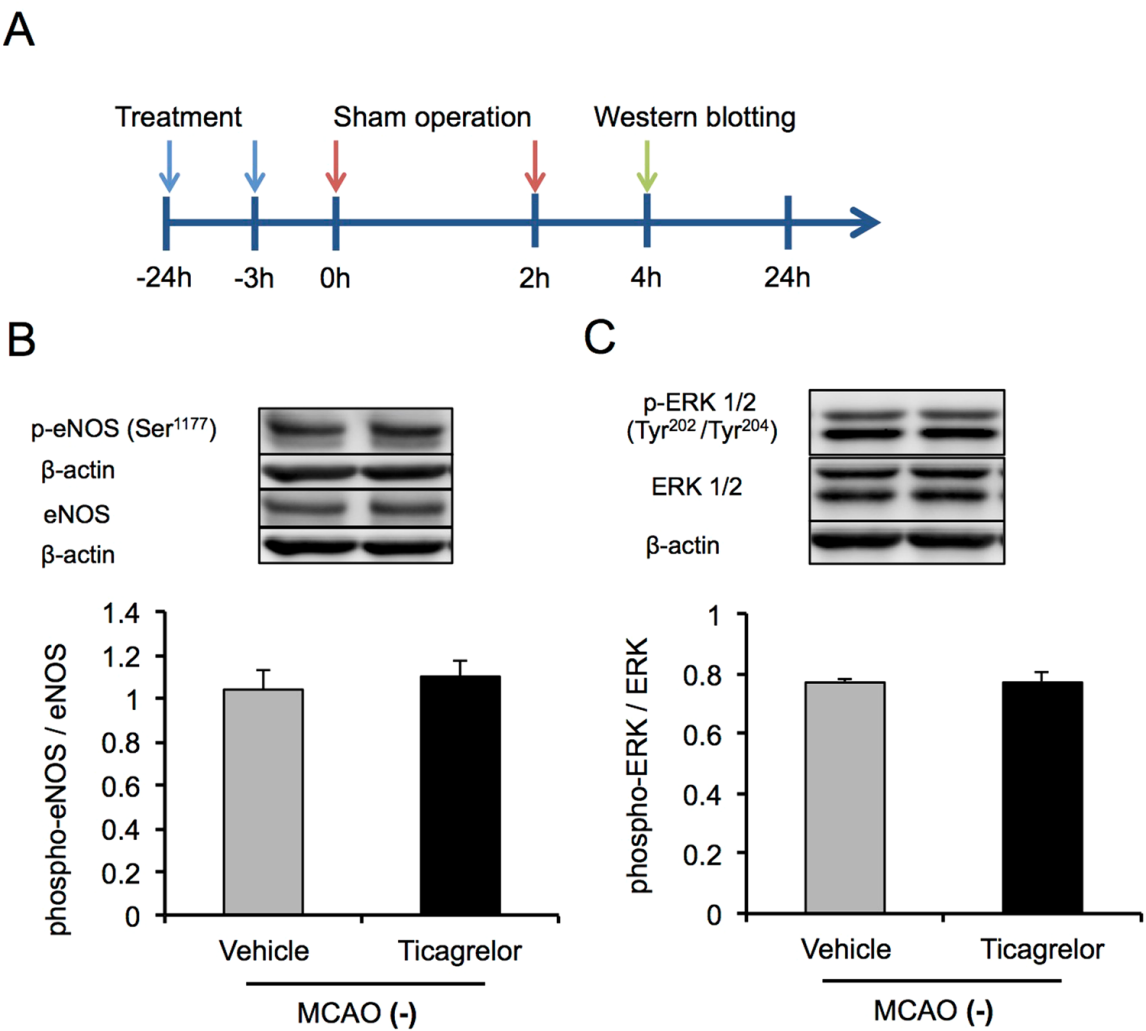
Effects of ticagrelor in sham-operated mice. (**A**) The experimental protocol used for western blotting. The effects of ticagrelor on the phosphorylation of endothelial nitric oxide synthase (**B**) and extracellular signal-regulated kinase 1/2 (**C**) in sham-operated mice. Data are expressed as means ± standard errors of the mean. N = 5 per group. The cropped blots are used in this Figure and the full-length blots are presented in Supplementary Figure S3.

To investigate the mechanisms underlying the protective effects of ticagrelor against IRI, western blotting was conducted at 2, 6, and 22 hours after reperfusion using peri-infarct brain tissues (Fig. 4A). Thirty mg/kg of ticagrelor promoted the phosphorylation of endothelial nitric oxide synthase (eNOS) during the early phase after reperfusion, while the phosphorylation of eNOS was observed 24 hours after reperfusion in the other groups (Fig. 4A–C). In addition, 30 mg /kg of ticagrelor promoted the phosphorylation of extracellular signal-regulated kinase 1/2 (ERK1/2) two hours after reperfusion (Fig. 5). To investigate whether phosphorylation of eNOS and ERK1/2 related to pathological conditions, we evaluated the phosphorylation of eNOS and ERK1/2 also in sham-operated mice (Fig. 6A). Under non-pathological condition, 30 mg/kg of ticagrelor didn’t promote the phosphorylation of eNOS (Fig. 6B) and ERK1/2 (Fig. 6C).

### Effects of Ticagrelor on the *in vitro* OGD Stress Model

**Figure 7:**
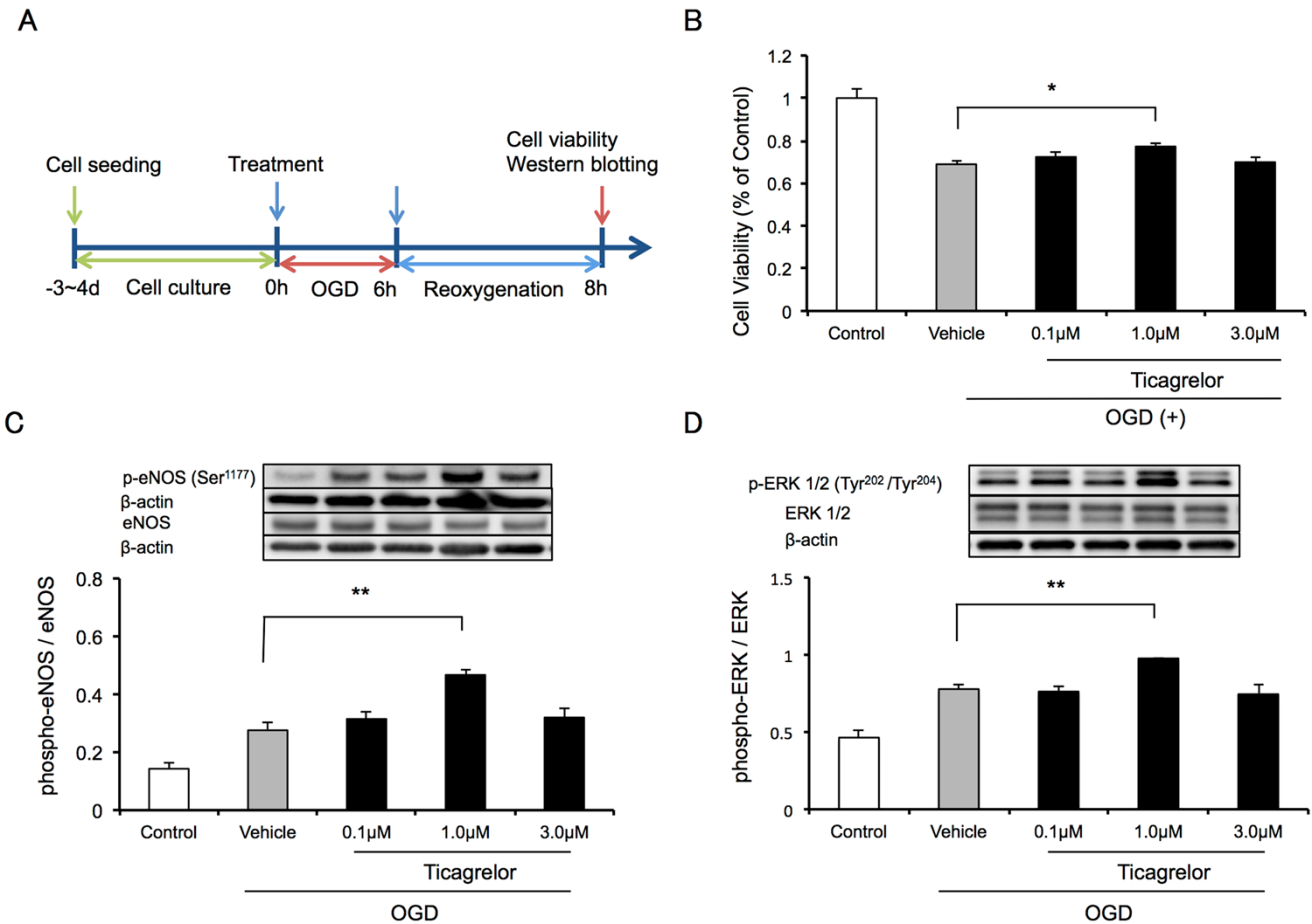
*In vitro* analysis using oxygen-glucose deprivation (OGD) stress in human brain microvessel endothelial cells (HBMVECs). (**A**) The experimental protocol used to assess the effects of ticagrelor against OGD stress in HBMVECs. (**B**) Cell viability measured using cell counting kit 8 two hours after reoxygenation. Data are expressed as means ± standard errors of the mean (SEMs). N = 12 per group; ^#^P < 0.05 vs. vehicle; Dunnett’s test. The effects of ticagrelor on the phosphorylation of endothelial nitric oxide synthase (**C**) and extracellular signal-regulated kinase 1/2 (D) 2 hours after reoxygenation. Data are expressed as means ± SEMs. n = 4 per group; ^##^P < 0.01 vs. vehicle; Dunnett’s test. The cropped blots are used in this Figure and the full-length blots are presented in Supplementary Figure S4.

To further investigate the mechanisms underlying the protective effects of ticagrelor, we investigated the effects of ticagrelor in an *in vitro* OGD stress model using HBMVECs. The protocol used in this study is illustrated in Fig. 7A. One μmol/L of ticagrelor improved cell viability 2 hours after reoxygenation when compared to vehicle treatment (Fig. 7B). Consistent with the results of the *in vivo* study, 1 μmol/L of ticagrelor promoted the phosphorylation of eNOS and ERK1/2 two hours after reoxygenation (Fig. 7C,D).

## Discussion

In this study, we evaluated the effects of ticagrelor against IRI *in vivo* and *in vitro*. We showed that the P2Y12 antagonist ticagrelor reduces infarct volume and improves outcomes after IRI in mice. We investigated ADP-induced platelet aggregation via oral gavage of ticagrelor (at −24 and −3 hours) to confirm its pharmacological activity. Ticagrelor inhibited platelet aggregation at 10 and 30 mg/kg. These data are consistent with those from previous reports indicating that ticagrelor inhibits platelet aggregation^7,8^. We also investigated the effects of aspirin as a control antiplatelet drug. The dose of aspirin was determined based on previous reports that investigated the inhibition of collagen-induced platelet aggregation by aspirin^9^. Ticagrelor at 3 mg/kg has been reported to have neuroprotective effects in a permanent MCAO rat model via inhibition of P2Y12 depending microglial inhibition^8^. Importantly, at this dose, ticagrelor did not inhibit platelet aggregation in the same rat study. Here we tested the effects of aspirin and ticagrelor in a mouse model of cerebral IRI at doses providing inhibition of platelet aggregation. Ticagrelor at 30 mg/kg, but not 10 mg/kg, nor 60 mg/kg of aspirin significantly reduced the infarct volume and improved neurological deficits 24 hours after the induction of MCAO, as shown in Fig. 1.

We investigated the putative mechanisms underlying the protective effects of ticagrelor other than its antiplatelet effects. We found that ticagrelor at 30 mg/kg induces the phosphorylation of eNOS 2 to 24 hours after the induction of MCAO when compared to control group. NOS catalyzes the oxidation of L-arginine and produces NO and L-citrulline^10^. NOS has three isoforms: eNOS, inducible NOS, and neuronal NOS (nNOS). Among these isoforms, eNOS is constitutively expressed in the vascular endothelium and the choroid plexus. Therefore, we confirmed the promotion of phosphorylation of eNOS after ischemia by ticagrelor in *in vitro* analysis using HBMVECs under a clinical relevant condition. This result suggests that ticagrelor affects endothelial cells directly. P2Y12 receptor is expressed in platelets and microglia, therefore our results suggest that ticagrelor has P2Y12-independent neuroprotective effects.

NO derived from eNOS plays an important role in preserving and maintaining the brain’s microcirculation by inhibiting platelet aggregation, leukocyte adhesion and migration, and reducing smooth muscle proliferation^11–23^, while NO derived from nNOS leads to neurotoxicity after cerebral ischemia^12–20^. The phosphorylation of eNOS at Ser^1177^ is one of the key regulators of its activity^13,25^. The phosphorylation of eNOS may be induced by several factors, including mechanical shear stress^13^, estrogen^14^, and vascular endothelial growth factor^15^. In addition, simvastatin (a 3-hydroxy-3-methylglutaryl coenzyme A reductase inhibitor) is reported to induce the phosphorylation of eNOS pharmacologically^16^.

Several authors have reported protective effects of eNOS modulation against IRI. Atochin *et al*. have reported that transgenic mice expressing phosphomimetic forms of eNOS display greater vascular reactivity, suppression of stroke, and improvements in CBF after 1 hour of transient MCAO when compared to transgenic mice expressing unphosphorylatable forms of eNOS^17^. Several agents, such as Rho kinase inhibitors^18^ and tetramethylpyrazine analogues^19^ have been reported to ameliorate brain damage after MCAO via pharmacological phosphorylation of eNOS. In contrast, pharmacological or genetic inhibition of eNOS exacerbates brain damage after cerebral ischemia^20,21^. These reports support the idea that phosphorylated eNOS plays an important role in the protection against ischemic brain injury. In this study, ticagrelor induced phosphorylation of eNOS at an earlier time than endogenous phosphorylation, which occurs 6 to 24 hours after cerebral IRI^22^. We also measured CBF 24 hours after the induction of MCAO. Thirty mg/kg (p.o.) of ticagrelor improved CBF 24 hours after induction of MCAO.

Since phosphorylation of ERK1/2 is associated with the phosphorylation of eNOS, we measured ERK1/2 phosphorylation levels 2 hours after reperfusion in the ticagrelor-treated group in our *in vivo* analysis. Ticagrelor upregulated the phosphorylation of ERK 1/2 in our *in vitro* study. ERK1/2 is a member of the mitogen-activated protein kinase family and exists mainly in the cytoplasm. Although the role of ERK1/2 in the setting of stroke is debatable, it is involved in the neuroprotective effects of growth factors, estrogen, preconditioning, and hypothermia^23^. There are several reports regarding the association between ERK 1/2 activation and eNOS. Ho *et al*. reported that adenosine induces phosphorylation of eNOS via ERK 1/2 phosphorylation in the nucleus tractus solitarii and modulates cardiovascular function in rats^24^. Cheng *et al*. reported that simvastatin induces eNOS up-regulation via the Ras-mediated phosphatidylinositol 3-kinase-AKT and extracellular signal-regulated kinase (ERK)− 40 S ribosomal protein S6 kinase signaling pathways in the nucleus tractus solitarii and induces central hypotensive effects^25^. In previous *in vitro* studies using endothelial cells, activation of ERK has been shown to be involved in the phosphorylation of eNOS and the NO-mediated production of adenosine, estrogen, and bradykinin^14,26,27^. A recent study indicated that ticagrelor inhibits the cellular uptake of adenosine via ENT-1 and leads to an increase in the extracellular concentration of adenosine^4^. It is reported that exogenous adenosine increases coronary blood flow velocity in healthy volunteers taking 180 mg of ticagrelor orally^28^. It is also reported that intracerebral concentrations of adenosine are elevated under ischemic conditions^29^ and that pharmacological inhibition of adenosine uptake has neuroprotective effects against focal cerebral ischemia^30,31^. In myocardial IRI models ticagrelor has been shown to reduce infarct size via an adenosine mediated mechanism and to increase the phosphorylation of eNOS, ERK, Akt also via an adenosine mediated mechanism^5,32^. Taken together, the above data indicate that the activation of eNOS via ERK1/2 may underlie the neuroprotective effects of ticagrelor and that the inhibition of adenosine uptake by ticagrelor might be associated with these effects.

Ticagrelor is expected as a prophylactic agent for recurrence of ischemic events. Our results, ticagrelor has P2Y12-independent neuroprotective effects, suggests that ticagrelor has additional effects as a prophylactic agent for recurrence of ischemic events.

In conclusion, ticagrelor significantly improved cerebral blood flow, reduced infarct volume, and improved neurological function after cerebral IRI. The phosphorylation of eNOS and ERK 1/2 in endothelial cells may underlie the neuroprotective effects of ticagrelor. These findings indicate that ticagrelor has protective effects on cerebral IRI by mechanisms other than platelet inhibition.

## Material and Methods

### Animals

The experimental design and procedures of this study were approved by the animal experiment committees of Gifu Pharmacological University and were in compliance with Animal Research: Reporting *in Vivo* Experiments guidelines. Careful consideration was given to minimizing the numbers of animals used and their suffering. All *in vivo* experiments were conducted using male ddY mice (6–8 weeks old; body weight, 28–32 g) purchased from Japan SLC, Inc. (Hamamatsu, Shizuoka, Japan). All animals were kept in a controlled environment (24 ± 2 °C; 12-hour light/dark cycle) and permitted to access filtered clean water and standard laboratory food freely.

Two-hundred and eighty-six mice were used for this study. The number of mice used in each experiment is as follows: (1) platelet aggregation test, 48 mice; (2) MCAO model, 129 mice (12 mice excluded); (3) western blotting, 109 mice (17 mice excluded).

### Drug Treatment

Ticagrelor (LKT Laboratories, Inc., Saint Paul, Minnesota, USA) and aspirin (Cayman Chemical, Ann Arbor, Michigan, USA) were purchased. Mice were randomly divided into two ticagrelor treatment groups (10 mg/kg and 30 mg/kg), an aspirin treatment group (60 mg/kg), and a vehicle treatment group. Drugs were dissolved in 0.4 mL 1.0% carboxymethylcellulose and administrated by oral gavage at 3 and 24 hours before MCAO and 0 and 6 hours after reperfusion. Mice in vehicle treatment group were administered the same amount of carboxymethylcellulose at same time point.

### *Ex Vivo* Platelet Aggregation Test

Platelet aggregation was measured using light transmission aggregometry, as previously described^33^. Mice were treated 24 hours and 3 hours before the measurement of platelet aggregation. Under deep anesthesia with pentobarbital, whole blood was obtained by cardiac puncture using a 22-gauge needle. Four milliliters of whole blood obtained from four or five mice were collected in a 5-mL tube containing 3.0 mL of 3.8% sodium citrate. Platelet-rich plasma was obtained by centrifugation at 155 g for 12 minutes at room temperature. Twenty micromoles per liter adenosine diphosphate (Sigma-Aldrich Co., St. Louis, MO, USA) was added to 225 μL of platelet-rich plasma to induce platelet aggregation. Platelet aggregation was measured using an aggregometer (PA200 apparatus; Kowa Co., Ltd.; Tokyo, Japan). During the measurement of platelet aggregation, the samples were continuously stirred and kept at 37 °C.

### Focal Cerebral Ischemia Mouse Model

To induce focal ischemia, a mouse MCAO model was established, as previously described^34^. In brief, mice were anesthetized using a mixture of 70% NO_2_, 30% O_2_, and 2.0–2.5% isoflurane (Mylan, Canonsburg, PA, USA) using an animal general anesthesia machine (Soft Lander; Sin-ei Industry Co., Ltd., Saitama, Japan). The left common carotid artery was exposed using a midline skin incision. The origin of the middle cerebral artery was occluded using an 8–0 nylon monofilament coated with Provil novo (Heraeus, Hanau, Germany). After 2 hours of MCAO, the filament was removed to establish reperfusion under brief anesthesia. The body temperature of the mouse was kept at 37.0–37.5 °C using a heating lamp and a heating pad during the procedure. After the procedure, the mice were kept in the same conditions as the preoperative environment (24 ± 2 °C; 12-hour light/dark cycle) until further experiments.

The following exclusion criteria were set before the experiment: (1) death during surgery due to procedural or anesthetic problems; (2) death before sampling; (3) inability to achieve reperfusion; (4) absence of a detectable ischemic core; and (5) a reduction of CBF less than 30% of baseline, as measured using laser Doppler flowmetry (LSFG-ANW; Softcare Co., Ltd.; Fukuoka, Japan).

### Assessment of Cerebral Infarct

The experimenters (K.Y. and T.I.) performing the assessments were blinded to the experimental groups. Infarct volume was quantified as previously described^34^. In brief, the forebrains of mice were removed under deep anesthesia with isoflurane (Mylan; Canonsburg, PA, USA) 24 hours after the induction of MCAO. Five 2-mm thick coronal sections were stained using a 2% TTC (Sigma–Aldrich Co., St. Louis, MO, USA) solution for 30 minutes. The stained sections were digitally imaged and analyzed using image-processing software (Image-J ver. 1.43 h; National Institutes of Health, Bethesda, MD, USA).

### Behavioral Assessments

All behavioral tests were conducted in a quiet and low-light environment by blinded scientists (K.Y. and T.I.). As previously reported by Garcia *et al*., the following six behaviors were assessed: (1) spontaneous activity for 5 minutes, (2) symmetry of the four limbs during movement, (3) symmetry of forelimb outstretching, (4) climbing ability, (5) body proprioception, and (6) response to vibrissae touch. Each behavior was scored on a scale between 0 and 3 points and a total score was calculated. Lower scores indicated more severe neurological deficits^35^. As previously reported by Lee *et al*., the grid walk test was performed to evaluate walking skill. Mice were allowed to freely walk on a grid (0.24 mm-wide wires, 10 mm^2^ openings), and the number of step errors was counted for 2 minutes^36^.

### Measurement of Cerebral Blood Flow

Periprocedural cerebral blood flow was measured using laser Doppler flowmetry (LSFG-ANW; Softcare Co., Ltd.; Fukuoka, Japan), as previously reported^37^. Mice were placed in the prone position under brief anesthesia with 2.0–3.0% isoflurane (Mylan; Canonsburg, PA, USA), and the skull was exposed using a linear skin incision. The skull was exposed to a 780-nm semiconductor laser. The reflected light, which was linearly polarized, was detected using a charge-coupled device camera placed above the head through a hybrid filter. The hybrid filter was used to preclude the detection reflected light from the surface of the tissue and to enable stable and specific measurements. The raw speckle images were videotaped to evaluate the speckle contrast, which indicates the velocity and numbers of moving red blood cells. The average of 20 consecutive raw speckle images was used to obtain one blood flow image. The red color indicates the higher cerebral blood flow. The images were analyzed using the software installed in a laser speckle imaging system (Omegazone; Omegawave, Inc.; Tokyo, Japan). Oval regions of interest (ROIs) were created at the ipsilateral and contralateral middle cerebral artery (MCA) areas. Relative blood flow in the MCA area was calculated by averaging the blood flow of the ROIs, and the ratio of ipsilateral CBF to contralateral CBF was calculated. CBF was measured just before MCAO, just after MCAO, just before reperfusion, just after reperfusion, and 4 and 22 hours after reperfusion.

### *In vitro* Oxygen-Glucose Deprivation Model

To assess the effects of ticagrelor against IRI in endothelial cells *in vitro*, we used an OGD model in HBMVECs, as previously reported^38^. Briefly, the cells were cultured in 96-well culture plates until they became confluent. The cells were then washed twice with glucose-free Dulbecco’s modified Eagle’s medium (DMEM) and incubated in the same glucose-free medium in an oxygen-free incubator (94% N_2_, 5% CO_2_, and 1% O_2_) for 6 hours, and under normal growth conditions for an additional 2 hours (reoxygenation). Ticagrelor (0.1, 1.0, and 3.0 μmol/L) was added to the medium just before OGD and just after reoxygenation. The concentration of ticagrelor was determined according to the Cmax (1.4 ± 0.7 (μmol/L ± SD)) and Cmin (0.4 ± 0.7 (μmol/L ± SD)) levels in patients taking 4 weeks of standard doses of 90 mg BID as previously reported^39^. Control cells were incubated in DMEM. We performed the cell viability and western blot assays 2 hours after reoxygenation.

Cell viability after OGD was measured using Cell Counting Kit 8 (Dojindo Molecular Technologies, Inc.) as previously described^40^. Two hours after reoxygenation, the cells were incubated with 10% 2-(2-methoxy-4-nitrophenyl)−3-(4-nitrophenyl)-5-(2,4-disulfophenyl)-2H-tetrazolium, monosodium salt for 3 hours at 37 °C, and optical density at 450 nm was measured using a microplate reader (Varioskan Flash 2.4; Thermo Fisher Scientific; Waltham, MA, USA).

### Western Blotting

The forebrains of mice were removed quickly under deep anesthesia with isoflurane 2 hours, 6 hours, and 22 hours after reperfusion. Tissue from the peri-infarct area was also collected. In the cell culture experiments, the cells were collected 2 hours after reoxygenation. Ten milliliters ice-cold radioimmunoprecipitation assay lysis buffer and protease/phosphatase inhibitor per gram of tissue was used for homogenization (Sigma-Aldrich Co.). After centrifugal separation (12,000 × *g* for 30 minutes at 4 °C), supernatants from the samples were collected. Ten micrograms of the protein samples were applied onto 5–20% gradient sodium dodecyl sulfate polyacrylamide gels (SuperSep Ace; Wako Pure Chemicals; Osaka, Japan) and separated according to molecular weight using electrophoresis. The separated proteins were transferred to polyvinylidene fluoride membranes (Immobilon-P; Millipore Corporation; Billerica, MA, USA). The following primary antibodies were used: rabbit anti-phosphorylated eNOS Ser1177 (1:1,000; Cell Signaling Technology; MA, USA), rabbit anti-eNOS (1:200; Santa Cruz Biotechnology, Inc.; TX, USA), rabbit anti-phospho-ERK1/2 Thr202/Tyr204 (1:1,000, Cell Signaling Technology), rabbit anti ERK1/2 (1:1,000, Cell Signaling Technology), and monoclonal anti-β-actin (1:2,000; Sigma-Aldrich Co. LCC, MO, USA). The following secondary antibodies were used: anti-rabbit immunoglobulin G (IgG) (1:1,000; Thermo Scientific; MA, USA) and anti-mouse IgG (1:1,000; Pierce Biotechnology; Rockford, IL, USA). Immunoreactive bands were visualized using a Lumino imaging analyzer (LAS-4000; Fujifilm; Tokyo, Japan). Multi Gauge software (Fujifilm; Tokyo, Japan) was used to analyze differences in band intensity.

### Statistical analysis

All data are shown as means ± standard errors of the mean. We used Student’s two-paired *t*-tests for two-group comparison, Wilcoxon signed-rank tests to compare nonparametric values and one-way analysis of variance was followed by Dunnett’s test for multiple pair-wise comparisons. P values < 0.05 were considered statistically significant. All statistical data were analyzed using JMP ver. 11 software (SAS Institute, Inc.; Cary, NC, USA).

## Electronic supplementary material

Supplementary Information

## References

[1] Donnan GA, Fisher M, Macleod M, Davis SM (2008). Stroke. Lancet.

[2] Wallentin L (2009). Ticagrelor versus clopidogrel in patients with acute coronary syndromes. The New England journal of medicine.

[3] Schneider DJ (2011). Mechanisms potentially contributing to the reduction in mortality associated with ticagrelor therapy. Journal of the American College of Cardiology.

[4] Armstrong D (2014). Characterization of the adenosine pharmacology of ticagrelor reveals therapeutically relevant inhibition of equilibrative nucleoside transporter 1. Journal of cardiovascular pharmacology and therapeutics.

[5] Nanhwan MK (2014). Chronic treatment with ticagrelor limits myocardial infarct size: an adenosine and cyclooxygenase-2-dependent effect. Arteriosclerosis, thrombosis, and vascular biology.

[6] Johnston SC (2016). Ticagrelor versus Aspirin in Acute Stroke or Transient Ischemic Attack. The New England journal of medicine.

[7] Gelosa P (2014). Microglia is a key player in the reduction of stroke damage promoted by the new antithrombotic agent ticagrelor. Journal of cerebral blood flow and metabolism: official journal of the International Society of Cerebral Blood Flow and Metabolism.

[8] Sugidachi A (2013). A comparison of the pharmacological profiles of prasugrel and ticagrelor assessed by platelet aggregation, thrombus formation and haemostasis in rats. British journal of pharmacology.

[9] Takagi T (2015). Cilostazol ameliorates collagenase-induced cerebral hemorrhage by protecting the blood-brain barrier. Journal of cerebral blood flow and metabolism: official journal of the International Society of Cerebral Blood Flow and Metabolism.

[10] Stuehr DJ, Griffith OW (1992). Mammalian nitric oxide synthases. Advances in enzymology and related areas of molecular biology.

[11] Toda N, Ayajiki K, Okamura T (2009). Cerebral blood flow regulation by nitric oxide: recent advances. Pharmacological reviews.

[12] Huang Z (1994). Effects of cerebral ischemia in mice deficient in neuronal nitric oxide synthase. Science.

[13] Dimmeler S (1999). Activation of nitric oxide synthase in endothelial cells by Akt-dependent phosphorylation. Nature.

[14] Hisamoto K (2001). Induction of endothelial nitric-oxide synthase phosphorylation by the raloxifene analog LY117018 is differentially mediated by Akt and extracellular signal-regulated protein kinase in vascular endothelial cells. The Journal of biological chemistry.

[15] Scotland RS (2002). Functional reconstitution of endothelial nitric oxide synthase reveals the importance of serine 1179 in endothelium-dependent vasomotion. Circ Res.

[16] Kureishi Y (2000). The HMG-CoA reductase inhibitor simvastatin activates the protein kinase Akt and promotes angiogenesis in normocholesterolemic animals. Nat Med.

[17] Atochin DN (2007). The phosphorylation state of eNOS modulates vascular reactivity and outcome of cerebral ischemia *in vivo*. The Journal of clinical investigation.

[18] Yagita Y (2013). Functional deterioration of endothelial nitric oxide synthase after focal cerebral ischemia. Journal of cerebral blood flow and metabolism: official journal of the International Society of Cerebral Blood Flow and Metabolism.

[19] Yan S (2015). Tetramethylpyrazine analogue CXC195 ameliorates cerebral ischemia-reperfusion injury by regulating endothelial nitric oxide synthase phosphorylation via PI3K/Akt signaling. Neurochemical research.

[20] Huang Z (1996). Enlarged infarcts in endothelial nitric oxide synthase knockout mice are attenuated by nitro-L-arginine. Journal of cerebral blood flow and metabolism: official journal of the International Society of Cerebral Blood Flow and Metabolism.

[21] Greco R (2011). IkappaB-alpha expression following transient focal cerebral ischemia is modulated by nitric oxide. Brain research.

[22] Osuka K (2004). Modification of endothelial NO synthase through protein phosphorylation after forebrain cerebral ischemia/reperfusion. Stroke; a journal of cerebral circulation.

[23] Sawe N, Steinberg G, Zhao H (2008). Dual roles of the MAPK/ERK1/2 cell signaling pathway after stroke. Journal of neuroscience research.

[24] Ho WY (2008). Adenosine modulates cardiovascular functions through activation of extracellular signal-regulated kinases 1 and 2 and endothelial nitric oxide synthase in the nucleus tractus solitarii of rats. Circulation.

[25] Cheng WH (2013). Simvastatin induces a central hypotensive effect via Ras-mediated signalling to cause eNOS up-regulation. British journal of pharmacology.

[26] Bernier SG, Haldar S, Michel T (2000). Bradykinin-regulated interactions of the mitogen-activated protein kinase pathway with the endothelial nitric-oxide synthase. The Journal of biological chemistry.

[27] Wyatt AW (2002). Early activation of the p42/p44MAPK pathway mediates adenosine-induced nitric oxide production in human endothelial cells: a novel calcium-insensitive mechanism. FASEB journal: official publication of the Federation of American Societies for Experimental Biology.

[28] Wittfeldt A (2013). Ticagrelor enhances adenosine-induced coronary vasodilatory responses in humans. Journal of the American College of Cardiology.

[29] Matsumoto K, Graf R, Rosner G, Shimada N, Heiss WD (1992). Flow thresholds for extracellular purine catabolite elevation in cat focal ischemia. Brain research.

[30] Matsumoto K, Sakaki T, Kohmura E, Hayakawa T, Yamada K (1996). Amelioration of ischemic brain damage by the preischemic administration of propentofylline (HWA285) in a rat focal ischemia. Brain research.

[31] Johnson MP, McCarty DR, Chmielewski PA (1998). Temporal dependent neuroprotection with propentofylline (HWA 285) in a temporary focal ischemia model. European journal of pharmacology.

[32] Ye Y (2015). Ticagrelor protects the heart against reperfusion injury and improves remodeling after myocardial infarction. Arteriosclerosis, thrombosis, and vascular biology.

[33] Ishiguro M (2010). Phosphodiesterase-III inhibitor prevents hemorrhagic transformation induced by focal cerebral ischemia in mice treated with tPA. PloS one.

[34] Hara H (1997). Inhibition of interleukin 1beta converting enzyme family proteases reduces ischemic and excitotoxic neuronal damage. Proceedings of the National Academy of Sciences of the United States of America.

[35] Garcia, J. H., Wagner, S., Liu, K. F. & Hu, X. J. Neurological deficit and extent of neuronal necrosis attributable to middle cerebral artery occlusion in rats. Statistical validation. *Stroke; a journal of cerebral circulation* **26**, 627–634; discussion 635 (1995). 10.1161/01.str.26.4.627 7709410

[36] Lee S, Ueno M, Yamashita T (2011). Axonal remodeling for motor recovery after traumatic brain injury requires downregulation of gamma-aminobutyric acid signaling. Cell death & disease.

[37] Ogishima H (2011). Ligation of the pterygopalatine and external carotid arteries induces ischemic damage in the murine retina. Investigative ophthalmology & visual science.

[38] Ishiguro M (2011). Blockade of phosphodiesterase-III protects against oxygen-glucose deprivation in endothelial cells by upregulation of VE-cadherin. Current neurovascular research.

[39] Storey RF (2007). Inhibition of platelet aggregation by AZD6140, a reversible oral P2Y12 receptor antagonist, compared with clopidogrel in patients with acute coronary syndromes. Journal of the American College of Cardiology.

[40] Ogawa K (2014). Protective effects of bilberry and lingonberry extracts against blue light-emitting diode light-induced retinal photoreceptor cell damage *in vitro*. BMC complementary and alternative medicine.

