# Supplementary material for: Effects of ticagrelor in a mouse model of ischemic stroke
Source: Sci Rep. 2017 Sep 21;7:12088. doi: 10.1038/s41598-017-12205-w (PMC5608945; doi:10.1038/s41598-017-12205-w)
Supplement: Supplementary file 1 — Supplementary Information [file 41598_2017_12205_MOESM1_ESM.pdf]

Effects of ticagrelor in a mouse model of ischemic stroke

Keita Yamauchi[1,2], Takahiko Imai[1], Masamitsu Shimazawa[1], Toru Iwama[2], Hideaki Hara[1*]

[1]Molecular Pharmacology, Department of Biofunctional Evaluation, Gifu Pharmaceutical University, Gifu, 501-1196, Japan.

[2]Department of Neurosurgery, Gifu University Graduate School of Medicine, Gifu, 501-1194, Japan.

*Corresponding Author:

Professor H. Hara, Ph.D., R.Ph., Molecular Pharmacology, Department of Biofunctional Evaluation, Gifu Pharmaceutical University, 1-25-4 Daigaku-nishi, Gifu 501-1196, Japan.

Tel & Fax: +81-58-230-8126

e-mail: hidehara@gifu-pu.ac.jp

Supplementary information

Western blotting

Detailed methods of western blotting are described in the main text.

Supplementary Figure 1.

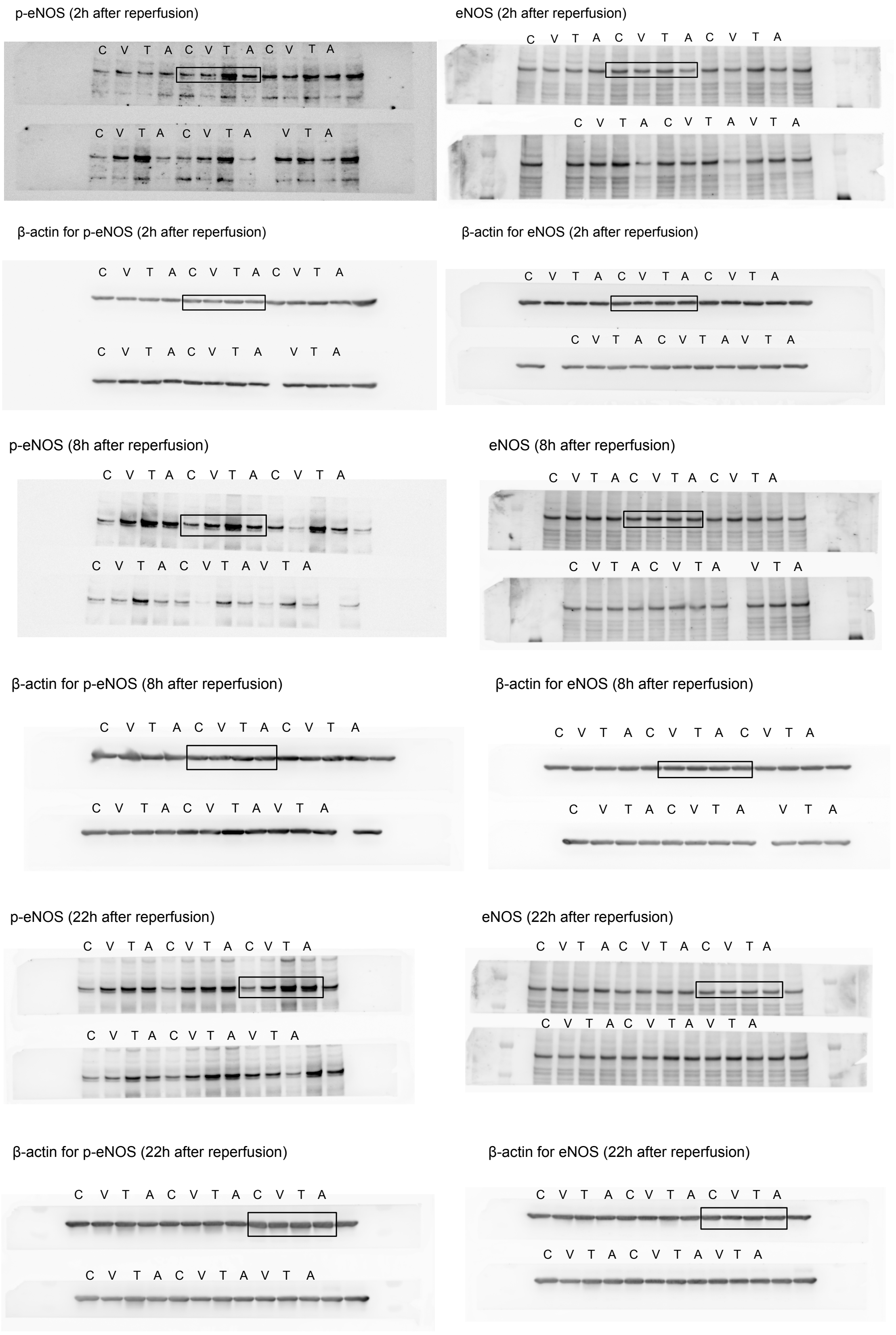

Figure S1 The full-length blots of western blotting in Figure 4.

C: control group; V: vehicle group; T: ticagrelor (30mg/kg) group; A: aspirin (60mg/kg) group.

Supplementary Figure 2.

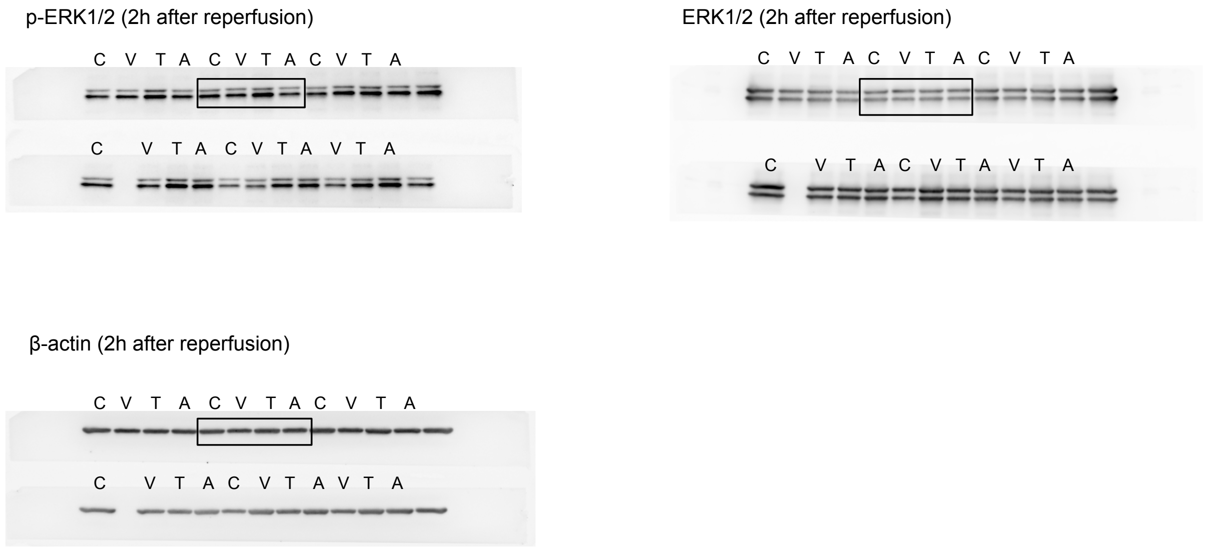

Figure S2 The full-length blots of western blotting in Figure 5.

C: control group; V: vehicle group; T: ticagrelor (30mg/kg) group; A: aspirin (60mg/kg) group.

Supplementary Figure 3.

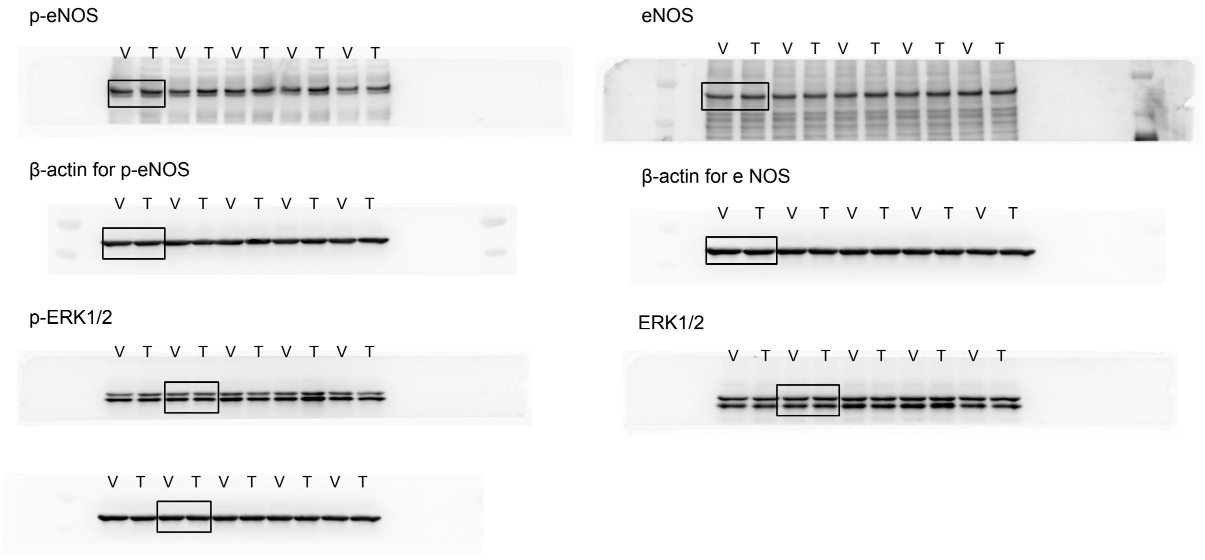

Figure S3 The full-length blots of western blotting in Figure 6.

V: vehicle group; T: ticagrelor (30mg/kg) group.

Supplementary Figure 4.

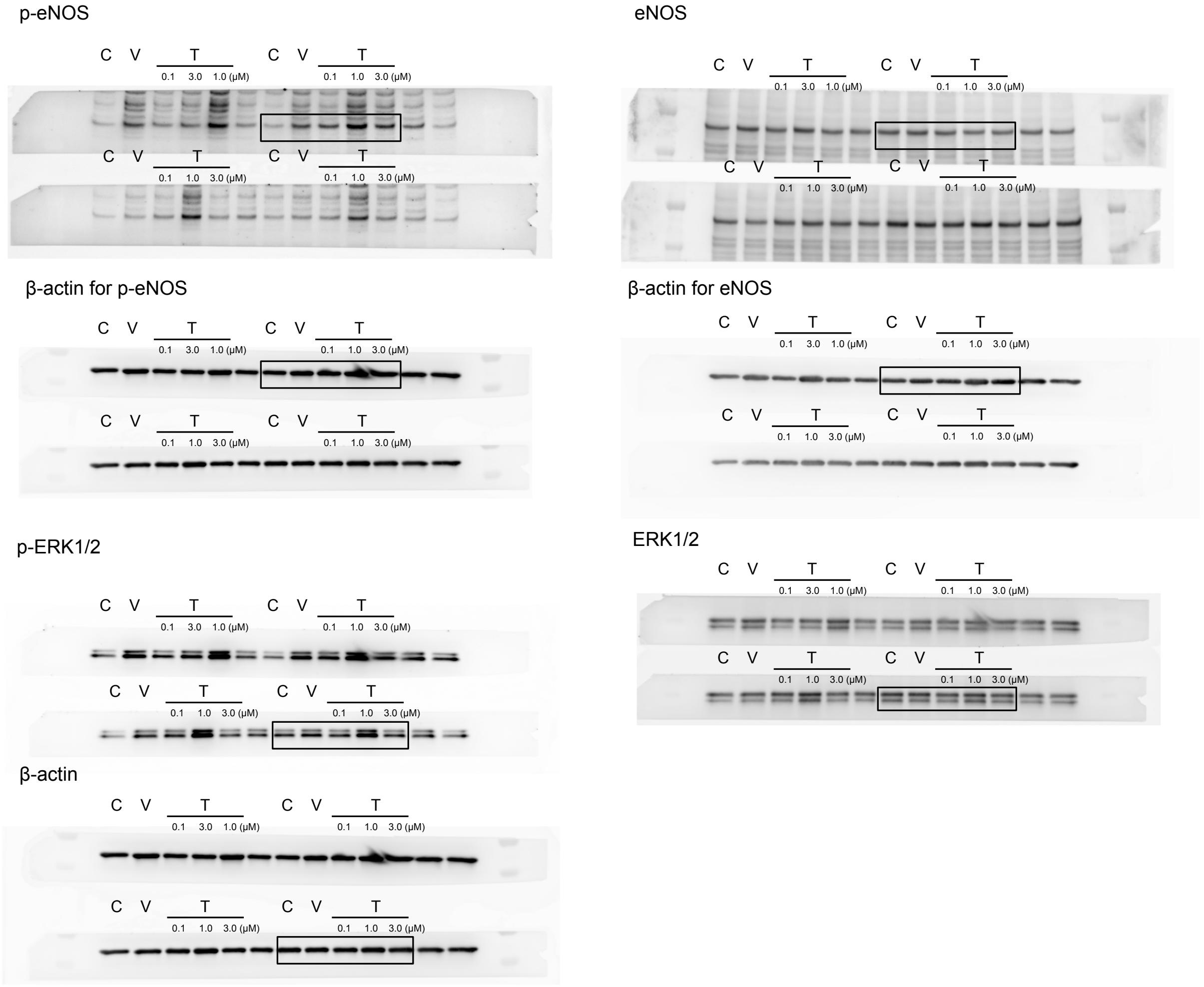

Figure S4 The full-length blots of western blotting in Figure 7.

C: control group; V: vehicle group; T: ticagrelor group.
